# A Comparison of Infectious Disease Forecasting Methods across Locations, Diseases, and Time

**DOI:** 10.3390/pathogens11020185

**Published:** 2022-01-29

**Authors:** Samuel Dixon, Ravikiran Keshavamurthy, Daniel H. Farber, Andrew Stevens, Karl T. Pazdernik, Lauren E. Charles

**Affiliations:** 1 Pacific Northwest National Laboratory, Richland, WA 99354, USA; samuel.dixon@pnnl.gov (S.D.); ravikiran.keshavamurthy@pnnl.gov (R.K.); daniel.farber@doh.wa.gov (D.H.F.); andrew.stevens@pnnl.gov (A.S.); karl.pazdernik@pnnl.gov (K.T.P.); 2 Paul G. Allen School for Global Health, Washington State University, Pullman, WA 99164, USA; 3 Department of Statistics, North Carolina State University, Raleigh, NC 27695, USA

**Keywords:** infectious disease forecasting, prediction, big data, multi-feature fusion, machine learning, deep learning, GLARMA, campylobacteriosis, typhoid, Q-fever

## Abstract

Accurate infectious disease forecasting can inform efforts to prevent outbreaks and mitigate adverse impacts. This study compares the performance of statistical, machine learning (ML), and deep learning (DL) approaches in forecasting infectious disease incidences across different countries and time intervals. We forecasted three diverse diseases: campylobacteriosis, typhoid, and Q-fever, using a wide variety of features (n = 46) from public datasets, e.g., landscape, climate, and socioeconomic factors. We compared autoregressive statistical models to two tree-based ML models (extreme gradient boosted trees [XGB] and random forest [RF]) and two DL models (multi-layer perceptron and encoder–decoder model). The disease models were trained on data from seven different countries at the region-level between 2009–2017. Forecasting performance of all models was assessed using mean absolute error, root mean square error, and Poisson deviance across Australia, Israel, and the United States for the months of January through August of 2018. The overall model results were compared across diseases as well as various data splits, including country, regions with highest and lowest cases, and the forecasted months out (i.e., nowcasting, short-term, and long-term forecasting). Overall, the XGB models performed the best for all diseases and, in general, tree-based ML models performed the best when looking at data splits. There were a few instances where the statistical or DL models had minutely smaller error metrics for specific subsets of typhoid, which is a disease with very low case counts. Feature importance per disease was measured by using four tree-based ML models (i.e., XGB and RF with and without region name as a feature). The most important feature groups included previous case counts, region name, population counts and density, mortality causes of neonatal to under 5 years of age, sanitation factors, and elevation. This study demonstrates the power of ML approaches to incorporate a wide range of factors to forecast various diseases, regardless of location, more accurately than traditional statistical approaches.

## 1. Introduction

Infectious diseases have a significant negative impact on veterinary and public health, both at the regional and global levels. The emergence of novel infectious agents, the reemergence of infectious agents previously under control, the constant burden of the endemic pathogens, and the development of antimicrobial resistance have complicated control and prevention initiatives. The threat posed by these diseases varies widely in terms of mortalities, morbidity, social, and economic disruptions. These threats are further magnified by anthropogenic and ecological factors, such as rapidly increasing population, globalization, urbanization, climate change, administrative conflicts, weak health systems, and the changing nature of pathogen transmission between human and animal populations [1]. Additionally, the wide variety of potential causes for such disease occurrences has made preparedness and timely response a challenge. Due to these reasons and despite a significant improvement in control and prevention efforts over the past few decades, infectious diseases continue to pose a major challenge, causing millions of deaths each year worldwide, especially in low-income countries [2]. 

Advanced knowledge of disease outbreaks can help public health decision-makers prevent, mitigate, and reduce infections and epidemic severity. Infectious disease forecasting aims at synthesizing epidemiological information related to the disease occurrence in advance for better allocating public health resources at appropriate places and times. Recent disease forecasting methods have taken advantage of the increasing abundance of publicly accessible data and advanced algorithms for better prediction of infectious disease outbreaks. These open access data streams are routinely updated, cover a wide variety of topics, and are increasingly detailed and complete, which could be used to increase the accuracy of the forecasting models [3,4]. 

Time series models have a long history of being used for disease forecasting. Traditionally, disease forecasting has relied on statistical techniques, such as autoregressive integrated moving average with exogenous variables (ARIMAX) and generalized linear autoregressive moving average models (GLARMA). These models are widely popular because they can be easily implemented and interpreted in retrospective studies. However, they can only process a limited amount of feature data and complexity to generate disease outcomes. Machine learning (ML) models, which have now been applied to a wide variety of time series regression tasks [5,6,7], are being used in disease forecasting to detect cryptic patterns arising from interactions between multiple features, which are difficult, and impossible at times, to uncover with conventional statistical methods. ML-based ensemble models, such as random forest (RF) and extreme gradient boost (XGB), are particularly popular in healthcare research because of their ease of implementation and interpretation [8,9]. These models are based on a decision tree approach that repeatedly splits the regression space non-linearly and can be visualized in the form of a tree. The RF uses an ensemble of hundreds of trees that are generated by bagging (random selection of a subset of data) and random resampling of a small number of features, while XGB makes use of a gradient boosting framework where trees are added sequentially to make predictions. These models have been successfully used in forecasting infectious diseases, such as infectious diarrhea [10], influenza [11,12], brucellosis [13,14], and dengue [15].

Deep learning (DL) models are ML models consisting of a minimum of one hidden layer of nodes between the input and output layer. A fully connected multi-layer perceptron (MLP) is the simplest architecture DL model, comprised of two or more layers of nodes, all of which are unidirectionally connected. A sequence-to-sequence encoder–decoder model (Enc–Dec) is a more complex architecture DL model, which consists of two subsections: the first subsection, called the encoder, translates a variable-length source input into a numerical embedding, and the second subsection, called the decoder, converts this numerical representation back to an output of the original form [16]. By creating this numerical vector representation as an intermediate, the Enc–Dec has been shown to better incorporate both short-term and long-term temporal features into forecasts [17]. DL models have outperformed ML models in several tasks, including time-series predictions [18]. However, DL models’ increase in predictive power relative to simpler ML models comes at a serious cost. DL algorithms are not readily interpretable, although there are recent advances in developing such techniques [19]. These models generally require more computational resources and experience to train [20,21] as well as more training data to generalize well [22].

In this study, we compare the performance of ML, DL, and statistical models to forecast three human infectious diseases: typhoid fever, Q-fever, and campylobacteriosis, across multiple geographic regions within Australia, Israel, and the United States. These diseases were selected because they are internationally notifiable diseases with disease cycles and prevalence rates that vary across regions and time, providing a broad range of scenarios to test the performance of the forecasting models. We demonstrate the utility of a traditional statistical time series model (GLARMA), ML regression trees (RF and XGB), and DL models (MLP and Enc–Dec) in forecasting diseases, thereby providing insights into how the models perform given different disease lifecycles and incidence across different geographic landscapes and climates as well as region-specific population demography and socioeconomic factors. Finally, we compare the performance of these disease models to accurately predict case counts at different times, from one month to eight months into the future.

## 2. Results

### 2.1. Datasets 

The mean infections of campylobacteriosis in 2009–2018 by month and region was greatest in Australia (i.e., 190 cases), nearly five times the next highest mean infection counts in the United States and Israel (Table 1). In addition, there were many more campylobacteriosis cases on average per month than either Q-fever or typhoid (range 0–4 mean cases/month) with Australia having the largest mean number of case counts for all of the explored diseases. There were more cases of Q-fever than typhoid in Australia and Israel, which was reversed in the US. None of the target countries showed a consistent seasonal disease pattern over the full time period, which increases the difficulty of forecasting. However, the differences observed in case counts and disease life cycles, as well as country features and incidence, enabled this study to investigate the performance advantages and disadvantages of the given forecasting methods under a variety of conditions.

**Figure 1:**
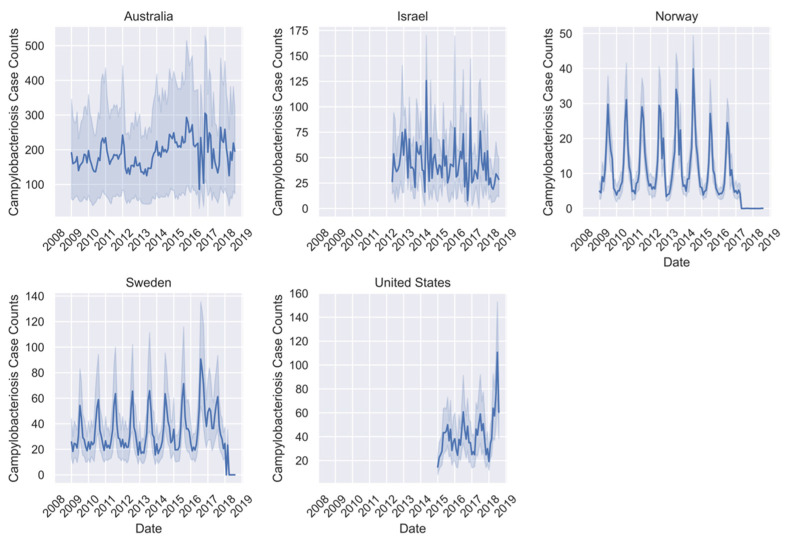
Mean campylobacteriosis case counts per region for available data between January 2009 and August 2018 for all seven countries with shaded areas representing two standard deviations from the mean. Note: only data from 2018 in Australia, Israel, and the United States was held out as the test set.

**Figure 2:**
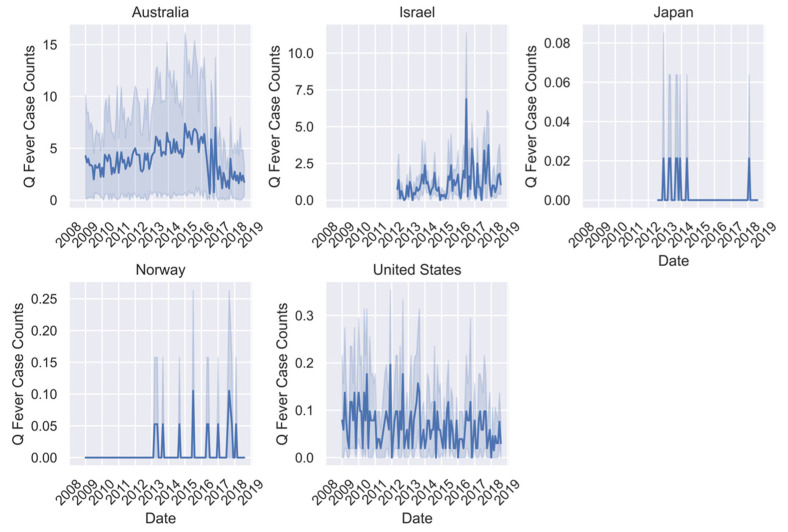
Mean Q-fever case counts per region for available data between January 2009 and August 2018 with shaded areas representing two standard deviations from the mean.

**Figure 3:**
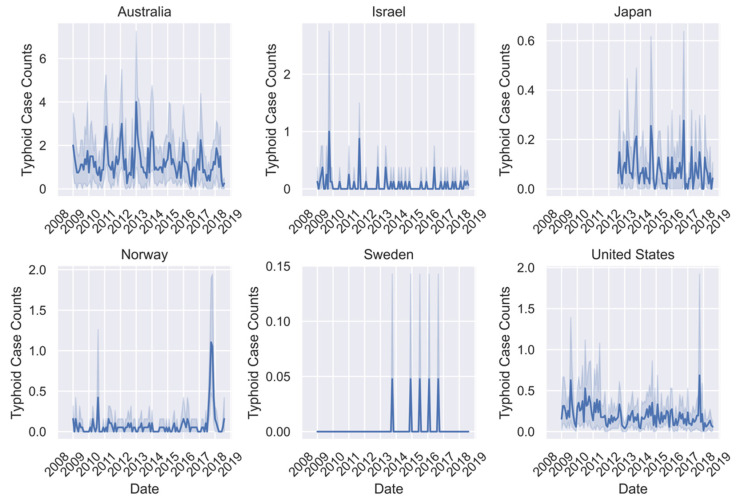
Time series of mean typhoid case counts per region for all available data between January 2009 and August 2018 with shaded areas representing two standard deviations from the mean.

All ML models were trained using the 2009–2017 data for all seven countries where available (Figure 1, Figure 2 and Figure 3). However, there was missing or erroneous data in 2018 for at least one disease in Finland, Japan, Norway, and Sweden. Therefore, models were evaluated for prediction performance on 2018 regional data only in Australia, Israel, and the United States. Australia is the only country where the full dataset of cases for all three diseases is present for the full time period. Israel and the United States are missing early years for campylobacteriosis, and Israel is also missing some for Q-fever. For all regions except Australia, there is a visual periodicity in average regional case counts of campylobacteriosis between 2009–2018 with a spike towards the end of each year (Figure 1). For Q-fever and typhoid, the annual pattern is less clear in part because of the low number of case counts (Figure 2 and Figure 3). 

### 2.2. Overall Model Performance 

**Figure 4:**
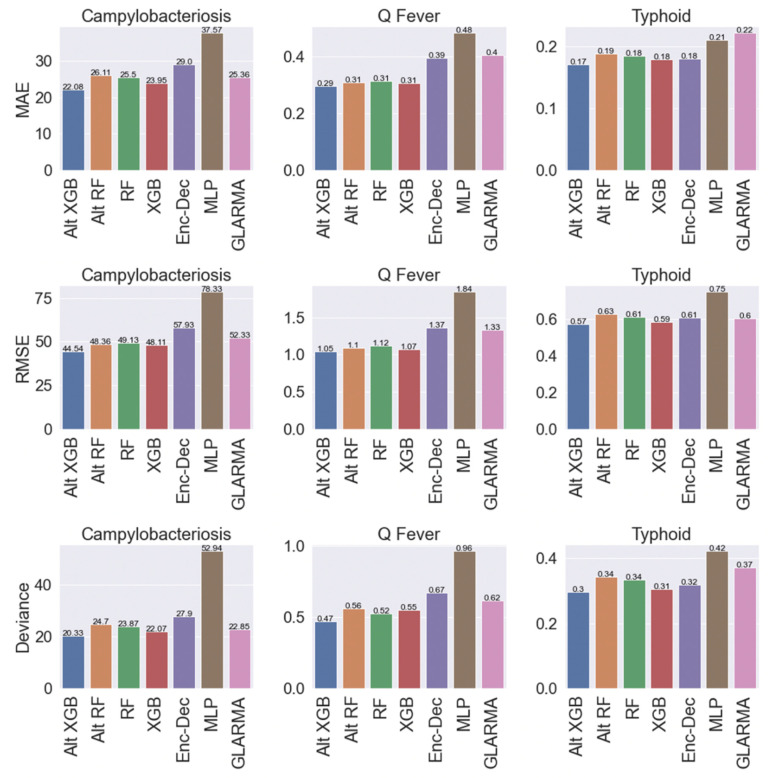
Average forecasting model performance by region for all predicted months during 2018 for campylobacteriosis, Q-fever, and typhoid as assessed by mean absolute error (MAE), root mean squared error (RMSE), and Poisson deviance (deviance) in Australia, Israel, and the United States.

The tree-based ML models (e.g., RF and XGB) were most accurate in forecasting campylobacteriosis, typhoid, and Q-fever than the DL or statistical models (Figure 4). The Alt-XGB (i.e., the XGB model without including country:region as a feature) outperformed all models for all diseases in MAE, RMSE, and Poisson deviance. On the other end of the spectrum, the MLP models performed the worst for all diseases and performance metrics. The statistical GLARMA model and DL Enc–Dec produced similar results across diseases and metrics, except MAE in typhoid where GLARMA performed the worst. Compared to the tree-based models, these models were in line for typhoid and minutely worse for the other diseases. The random forest models (RF and Alt-RF) had nearly identical performance across diseases and were only a few tenths away from the XGB models. The relative scale of the performance metrics is different between diseases; since the case counts are much higher all around for campylobacteriosis, the values ranged from 20.3–78.3, whereas the values for Q-fever (range 0.3–1.8) and typhoid (range 0.2–0.8) are much lower. Note that for the statistical time series models, the GLARMA model was fit with the negative binomial distribution and parameter estimation converged in all regions. For the tree-based ML models, the standard average pooling provided the best results out of all pooling methods attempted and, therefore, results are only shown using that method. 

### 2.3. Model Performance by Country

**Figure 5:**
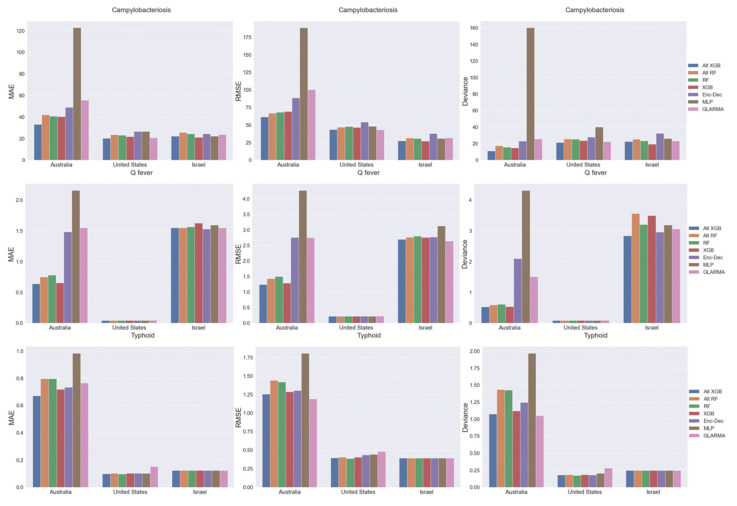
Mean performance of ML and statistical models by mean absolute error (MAE), root mean squared error (RMSE), and Poisson deviance (deviance) for Australia, United States, and Israel in forecasting monthly regional disease case counts in 2018.

The performance of each model by country and disease had similar overall performance trends regardless of metrics (Figure 5). For Australia, the MLP model was by far the worst performer. For campylobacteriosis and Q-fever, the tree-based models performed the best, notably the XGB (Alt and not) were the top performers. For typhoid, however, while XGB was best when assessed by MAE, the GLARMA model outperformed all other methods when assessed by RMSE and deviance. For the United States, the model performance results were all very close regardless of disease. Model performances were essentially the same for Q-fever; the tree-based models, namely RF, were slightly better for typhoid; and both Alt-XGB and GLARMA were slightly better for campylobacteriosis. For Israel, all models performed the same for typhoid. XGB slightly outperformed Alt-XGB as the top model for campylobacteriosis in Israel. Similarly, Q-fever performance in Israel depends on the metric used with Enc–Dec slightly better than Alt-XGB by MAE, GLARMA slightly better than Alt-XGB by RMSE, and Alt-XGB slightly better than Enc–Dec by deviance.

### 2.4. Model Performance by Time Interval

**Figure 6:**
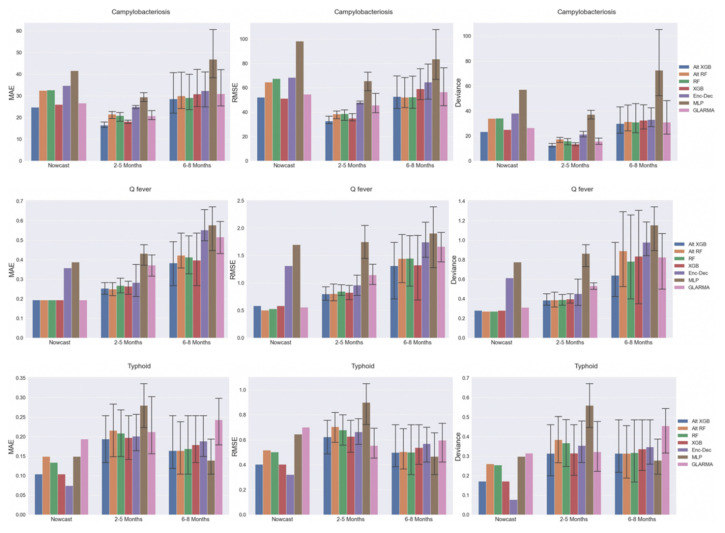
Comparison of model performance by metric in forecasting regional monthly case counts for January to August 2018 in Australia, Israel, and the United States. Error bar shows highest and lowest error by month.

The performance of the models by time interval (i.e., nowcast [1 month], short-term forecast [2–5 months], and long-term forecast [6–8 months]) varied by disease and location (Table 2; Figure 6). For nowcasting overall, XGB-based models performed the best for campylobacteriosis, RF-based for Q-fever, and Enc–Dec for typhoid. In general, for short-term forecast best models, XGB-based models were the best for campylobacteriosis, and all the tree-based models were comparable for Q-fever, whereas XGB (Alt and not) and/or GLARMA were best for typhoid based on metric. Namely, for typhoid, XGB and GLARMA performed similarly for deviance, Alt-XGB was best for MAE, and GLARMA for RMSE. For overall long-term forecast performance, there were more discrepancies in performance metrics. For campylobacteriosis, MAE and RMSE show the best models were RF (Alt and not) and Alt-XGB, whereas deviance favors nearly all models except MLP with Alt-XGB being minutely better. For Q-fever, Alt-XGB and XGB are comparable by MAE and RMSE with Alt-XGB performing the best by deviance. MLP, which performed the worst in any other circumstance, performed the best for long-term typhoid forecasting using all three metrics. When comparing relative performance across the time scales for the best performing model by disease, there are not any consistent patterns (Figure 6). For campylobacteriosis, short-term forecasting is most accurate followed by nowcasting, and then long-term forecasts. For Q-fever, the order of best to least performance is by time, i.e., nowcast, short-term forecasts, and then long-term forecasts. Typhoid, again different, has the best performance with nowcasting, then long-term forecasting, and lastly, short-term forecasting. In general, error bars are larger for long-term vs. short-term forecasting. 

### 2.5. Model Performance by Disease Incidence

**Figure 7:**
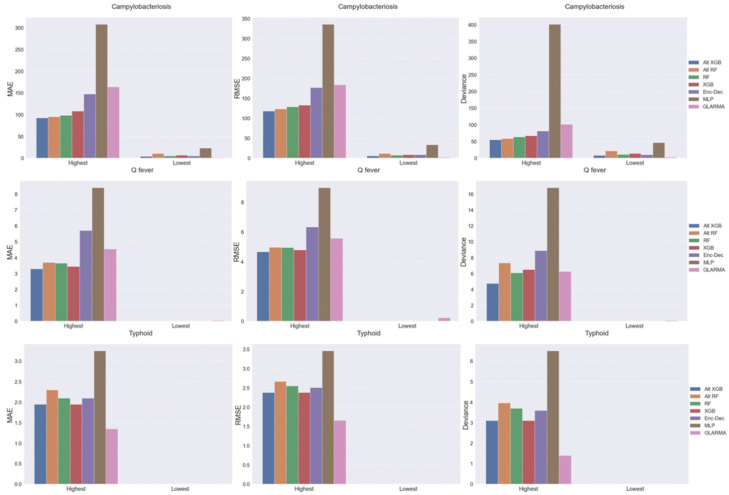
Comparison of model performance in forecasting monthly case counts from 2018 by metric for the 20 highest case count regions (highest) and lowest case count regions (lowest) across Australia, Israel, and the United States.

In 2018, across all regions in Australia, Israel, and the United States, there were a wide range of case counts from 0 to nearly 8000 per month. More specifically, the monthly case count ranges were 0 (25 regions) to 7742 (Queensland, Australia) for campylobacteriosis; 0 (10 regions) to 19 (Victoria, Australia) for typhoid; and 0 (62 regions) to 78 (Queensland, Australia) for Q-fever. The disease forecasting model performance increased with a decreasing number of cases, i.e., all models for the 20 regions with the lowest case counts outperformed those 20 regions with the highest case counts (Figure 7). For the regions with the lowest case counts, all models correctly predicted 0 case counts for typhoid and Q-fever, except GLARMA. However, for the lowest campylobacteriosis case counts, all models forecasted positive case counts in at least 15 of the 20 regions without a case count, with Alt-XGB and GLARMA performing the best and MLP performing the worst. For the highest case count regions, tree-based models (especially Alt-XGB) are optimal for campylobacteriosis and Q-fever, but GLARMA performs the best for typhoid. Overall, the DL models were the worst performers with regions that had high case counts. It is interesting to note that for counties that never had the disease, all models correctly predicted zero.

### 2.6. Summary of Top Model Performance by Disease

Overall, the top-performing models for forecasting campylobacteriosis, Q-fever, and typhoid vary by the way the resulting metrics are split (Table 3). For example, overall, the top-performing model for any disease, location, or forecasted month is Alt-XGB. However, when looking specifically at different data splits, other models outperformed or equally performed as well as Alt-XGB. 

### 2.7. Feature Importance

**Figure 8:**
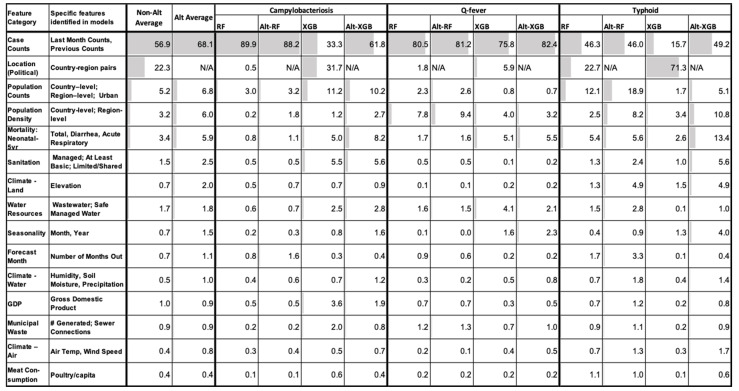
Top 20 features grouped in categories with their relative importance by disease and tree-based ML model-type shaded by contribution. Features are sorted by decreasing average relative importance of all Alt-models.

The relative importance of the 46 explanatory factors varied only slightly in each tree-based ML disease model (Figure 8). Greater than 90% of all model performance based on these features can be contributed (in descending order) to previous case counts, country-region for non-Alt models, population counts, population density, mortality of neonatal to under 5 years of age, and sanitation with elevation also included for Alt models. In general, RF (Alt and not) models tend to weigh the previous case counts most importantly for all diseases, whereas XGB equally uses location when predicting campylobacteriosis and almost five times as often as case counts for typhoid predictions. Whether or not the location is present, the top 20 features remain the same for all models with slight order variation depending on the exact model and disease. 

## 3. Discussion

Infectious diseases have the potential to cause significant veterinary, public health, and socioeconomic losses throughout the world. Hence, prior intelligence regarding their occurrences is crucial for their timely control and prevention. To the best of our knowledge, this is the first study comparing and forecasting the performance of ML, DL, and statistical models to forecast three notifiable human infectious diseases; typhoid, Q-fever, and campylobacteriosis for region-level predictions for three countries over three continents. 

*Datasets.* Since ML and DL models perform better with more training data, we used disease case count and feature data from seven countries to train our ML and DL models, while performing prediction on only the three countries with complete datasets. While all available data for the four other countries was used in training, none of these countries had consistent case count reporting and, in particular, had gaps in the forecasted year, 2018. By augmenting with empirical data, we boosted our training dataset numbers with observations of the diseases under different feature conditions without having to use simulation or imputation techniques that may or may not represent reality. The result was reasonably large training sets for each disease, with the largest set for campylobacteriosis, then typhoid, and lastly Q-fever.

*Model performance.* Averaged across all countries, months, and regions over eight months, the best ML forecasts outperformed conventional statistical approaches (i.e., GLARMA) by MAE, RMSE, and Poisson deviance for every disease. Comparing between the ML models, the tree-based models (i.e., XGB and RF) outperformed the DL models (i.e., Dec–Enc and MLP) by all metrics as well, with the XGB-based models routinely providing the best performance. Averaging performance within different subsets of the data (by country, time interval, or disease incidence) sometimes provided conflicting results on which methodology performed best. However, a general pattern was observed, with the number of conflicting metric results increasing as disease incidence decreased. In only one instance each, regardless of the metric, the GLARMA model, for regions with the highest incidence of typhoid, and the Enc–Dec, for nowcasting of typhoid, performed the best. But even in these scenarios, the difference in error metrics between these models and the best tree-based ML model was extremely small. In addition, the corresponding error metrics were so low in magnitude that the practical implications of using one model over the other are minimal. In summary, if a single methodology is preferred for simplicity, our results suggest that XGB-based models are the best option across the study diseases and locations.

*Model comparison.* Tree-based ML models and statistical approaches are interpretable in terms of the relative importance each explanatory variable plays in prediction. However, the ML models each demonstrate significant advantages over the statistical approach. These models can incorporate more exogenous variables and more complex interactions between variables than the univariate GLARMA, which resulted in improved predictions for the ML models. While the GLARMA model can incorporate some explanatory variables and their interactions, they require manual construction and pruning, which can become unmanageable as the number of features grows large. While DL models can also accommodate many explanatory variables, they generally performed the worst and the importance of the variables are not interpretable. The DL approaches here could have suffered from overfitting due to a variety of potential causes, most common being learning the noise of the training data [23]. DL models generally perform better with more data and, therefore, the Enc–Dec model may be able to outperform the ML approaches with more training data and/or greater temporal and/or spatial extents of the data. However, simple interpretation of the model via variable importance still remains a benefit of the ML tree-based models over the DL models. Given the evidence of DL models outperforming ML in other applications [24] and recent developments in hierarchical DL models, in which basal features are learned and then inform higher-level classification [25], there remains the potential for hierarchical DL to improve performance in disease forecasting while maintaining interpretability.

*Feature importance.* Across all diseases and models, the most significant feature contributing to tree-based ML model forecasting is previous case counts, which emphasizes the criticality of timely, reliable, and accurate disease surveillance data. The date, number of months forecasting out, and country-region location are all very accessible information that also play key roles in prediction. While the country-region location was critical to the non-Alt models, the fact that the Alt models did, on average, perform better suggests that the other features that were included were sufficient to predict future case counts. Notably, when the country-region location had a high degree of importance, it often was accompanied by a reduction in relative importance of previous case counts. These features are often correlated, as certain regions historically have a higher or lower incidence of different diseases. Comparing the RF and XGB models, country/region was more important to the XGB models which could be due to its greedy iterative approach of tree construction. At a high-level, the next most important features for all diseases included population factors, neonatal mortality, sanitation metrics, and water resources. The other major contributors identified are climate, seasonality, livestock consumption, GDP, and waste. However, it is important to note that including correlated features splits the importance between these features potentially decreasing their true importance and making the final interpretation of results challenging. By grouping the individual features together at a high-level, we hope to overcome this issue to some extent. 

Campylobacteriosis, being one of the most common infectious disease of humans globally, is mainly transmitted through contaminated food and water [26]. Similarly, typhoid is an important public health concern transmitted largely via sewage contamination of food or water sources. Given the preponderance of food and water borne transmission of these diseases, it is not surprising that the most important features in forecasting, aside from case counts, were largely related to population factors and sanitation metrics. Furthermore, the typhoid tree-based models in comparison with Q-fever or campylobacteriosis tree-based models relied less on the previous case counts relative to other factors, suggesting that the typhoid model learned better from these additional features. Q-fever is an important zoonotic disease, which has the potential to cause large outbreaks in humans and livestock [27]. Q-fever has a complex disease cycle compared to other two diseases in our study and a range of risk factors associated with it. Additionally, the disease has been often neglected by health personnel, resulting in a poor understanding of its epidemiology [28,29]. Due to these reasons, Q-fever forecasting is a challenge, even in countries with good disease surveillance infrastructure. In our study, the previous case counts dominated the most important features in forecasting Q-fever, contrasted with the relatively wide range of contributing explanatory variables in forecasting typhoid and campylobacteriosis. Nevertheless, population, seasonality, health, and sanitation metrics were still among the most important features. Additionally, the majority of the disease forecasting models were more accurate when country/region was not incorporated (Alt model), suggesting the models were learning features of the regions rather than the regions themselves. 

This study has several strengths as it represents a significant advancement in disease forecasting in a few key respects. Firstly, this study engineered a single data set containing 46 explanatory variables spanning seven different countries both at the regional spatial scale and monthly temporal scale which could be used to create and evaluate forecasts for almost any disease and model type. Secondly, we applied several different ML models (RF, XGB, Enc–Dec, and MLP) and a statistical model (GLARMA) using the same feature inputs for three internationally notifiable diseases (campylobacteriosis, Q-fever, and typhoid), which have highly divergent epidemic processes and incidences. These models were compared to each other using three metrics, MAE, RMSE, and Poisson deviance, which provided slightly differing analyses of each model’s performance. Thirdly, we used an interpretable ML approach to determine the relative importance of each explanatory variable in predicting future incidences of each of the three diseases. The most important features were retrieved from the tree-based ML models with and without one-hot encoded country/region vectors (RF and XGB versus Alt-RF and Alt-XGB) to provide a perspective of which data are most predictive with and without knowledge of specific country/regions. The study also had many limitations. Many data sets were only available for a subset of the locations studied and were, therefore, not incorporated into the model. As more data becomes available, we expect the models we examined here could incorporate them readily and improve the performance. Additionally, these models could be applied to additional locations and at finer spatiotemporal resolutions. While this data set is novel in the breadth of the explanatory variables considered, these disease forecasting models were limited by the available biosurveillance data and would benefit greatly from improved diagnostics and reporting of each disease as there is relatively limited labeled data to build a training dataset. There is very likely a bias undercounting disease incidence in rural and impoverished regions with less access to the most accurate diagnostic tools [30,31]. This bias and underreporting underscore the countries included in this study, which are higher in many socioeconomic metrics as compared with the median country and compared with the countries with the highest incidences of these diseases. Additionally, a future direction would be to consider a spatial component, as nearby regions appear to potentially be linked.

In conclusion, we have demonstrated the efficacy of forecasting campylobacteriosis, Q-fever, and typhoid, three internationally notifiable diseases of significant public health impact, using ML, DL, and statistical models. Overall, we found the tree-based ML models to be more accurate in forecasting compared to DL and statistical autoregressive models. Previous case counts were among the most important features in forecasting all diseases, with population density and factors relating to clean water and sanitation also very important across diseases. This study demonstrates the application of ML approaches in forecasting disease, and the wide range of data that can be incorporated into such models. This work has the potential to greatly improve the lives of people living in these areas and the safety of travelers. These ML models, particularly the XGB model, can offer a significant improvement in predicting emerging outbreaks over traditional surveillance methods, as its predictions can be updated rapidly by retraining the model in a matter of hours as digital data streams are updated [32]. Further use of ML models may improve our ability to mitigate future epidemics and may aid our understanding of which combinations of factors are likely to lead to outbreaks.

## 4. Methods

### 4.1. Data Collection

This study focused on three human infectious diseases: campylobacteriosis, Q-fever, and typhoid. Case count data of each disease from 2009–2018 were collected from EpiArchive [33]. The regional data, across seven countries from 2009–2017, were used to build the models and the final model performances were evaluated on available 2018 data, which was consistent only from Australia, Israel, and United States through August (Table 4). 

Identification of potential explanatory variables was informed by a literature review of the diseases. Only publicly accessible data available for all countries of interest were considered and collected (Table 5). However, some variables contained missing data for some time periods within a region of interest. These data were imputed, as described in Section 4.3. 

### 4.2. Data Engineering

Relevant features were extracted from each data source for the countries of interest. This raw data contained different file types, organization, spatial extents, spatial resolutions, temporal extents, and temporal resolutions. Data were resampled to a uniform monthly temporal and regional-level spatial resolution using GDAL [34]. The ML algorithms required tabular data without missing values. In instances where feature data were not continuously available every month through 2017, missing values were linearly extrapolated from the last date available [35].

Separate training data matrices were engineered for each disease. To build these matrices, all feature data types were combined into a single data set, in which each row denoted a single disease/location/region/time bin with corresponding explanatory variables. To enable the use of region-country as a feature in ML algorithms, each region-country was one-hot encoded, i.e., the categorical variable is represented by several columns equal to the number of entries; the rows corresponding to a particular entry contain a “1” in that entry’s column and a “0” in all other entry columns [36].

### 4.3. Data Analysis

A separate model was created in python for each disease for each algorithm type: moving average models, tree-based ML models, and DL models, described below. The response variable of each model was disease case counts for the countries/regions and dates available (Table 2). The same explanatory variables were included as features for all diseases (Table 3). 

#### 4.3.1. Moving Average Models

Moving average models served as a baseline statistical time-series analysis. ARIMA and ARIMAX were considered but were not viable solutions for this dataset because the data did not meet the normality requirement and struggled to converge with many explanatory variables. However, GLARMA with a negative binomial distribution was robust and useful for comparison in this study.

#### 4.3.2. Tree-Based ML Models

RF [37] and XGB [38] are the ensemble, tree-based ML regression methods used in the analyses. Our XGB models were built using the XGBoost python package [39], while our tree-based models, evaluation and tuning methods were created using the scikit-learn framework [40]. Two variations of each of the disease’s model were included, one containing all explanatory features collected (Table 2), and a second model, “Alt,” which lacked the one-hot encoded country/region data. The Alt-XGB and Alt-RF models enabled the comparison of model performance in situations when the country/region data are unknown. The data were split into test and training subsets using nested K-fold time-series-split cross-validation with five folds. The models were retrained 10 times across each fold, varying the hyper-parameters. The final model was trained on all of the data through 2017 using the best hyper-parameters. 

Each tree-based ML model output generated 12 predictions for each region-month interval by generating predictions from data one to 12 months before the prediction interval. For example, 12 predicted case counts in Wisconsin (USA) for January 2018 were generated, each one based on all explanatory variables in Wisconsin from a single month from January 2017 to December 2017. To extract a single prediction for each region and month, these 12 predictions were pooled via four approaches: maximum prediction, minimum prediction, standard average and average weighted by the inverse of the number month forward being predicted to reflect a decrease in confidence in longer-term predictions. 

#### 4.3.3. DL Models

The MLP and Enc–Dec models were created using pytorch [41] and tuned using available data from years 2009–2016 and validated on the year 2017. Since the computational time required for an exhaustive search of all hyperparameters was unreasonable for the DL models, we choose to tune on learning rate and optimizer (namely, Adam and stochastic gradient descent). Different methods of regularization (i.e., dropout and batch normalization) and model architecture (i.e., depth and breadth) were also explored in the early stages of model development. The final models were trained on all of the available data from 2009–2017. The encoder portion of the Enc–Dec model uses a gated recurrent unit (GRU) model to create a vector representation of the previous year of data by processing it sequentially. The decoder then takes that representation as input to a separate GRU and sequentially decodes it into predictions for the next year. 

### 4.4. Metrics

Model performance was assessed by mean absolute error (MAE), root mean square error (RMSE), and by the Poisson deviance (deviance). Note that MAE and deviance are preferred metrics for skewed and highly discrete data, and that MAE, in particular, offers a more natural measure of average error which (unlike RMSE) is unambiguous [42]. However, we included all metrics (i.e., MAE, RMSE, and deviance) for completeness. 

The deviance statistic for the Poisson distribution from each disease/location/time case count prediction was computed and averaged over each disease. The aggregate score was calculated by first averaging over time, then region, and finally country. Each model’s performance was assessed in generating disease incidence predictions over three time intervals: nowcast, i.e., the proximal month after the training data (January 2018); short-term forecast, i.e., 2–5 months after the training data (February 2018–May 2018); and long-term forecast, i.e., 6–8 months after the training data (June 2018–August 2018). Additionally, each model’s performance was assessed within individual countries and within individual regions. 

## Figures and Tables

**Table 1:** The total case counts for each disease by country for 2009–2018, including mean, minimum, and maximum of the monthly regional cases. All available data through 2017 for the seven countries were included in the ML training sets but only highlighted countries were used in performance evaluations. NA means not available.

	Campylobacteriosis Case Counts			Q-Fever Case Counts			Typhoid Case Counts		
	Mean	Min–Max	Total	Mean	Min–Max	Total	Mean	Min–Max	Total
Australia	190.10	0–880	182,498	3.92	0–32	3768	1.17	0–14	1122
Israel	40.64	0–361	29,429	1.12	0–21	809	0.06	0–7	63
Japan	NA	NA	NA	NA	0–2	10	0.07	0–7	264
Norway	10.36	0–184	22,839	0.201	0–1	14	0.08	0-8	169
Sweden	31.55	0–427	16,380	NA	NA	NA	~0.00	0–1	5
United States	42.04	0–794	98,720	0.06	0–3	423	0.19	0–29	1251

**Table 2:** The top-performing disease model by location and forecast length. For the model, if all metrics match, one model is listed; if the best model by metric did not match, the cell contains ‘MAE; RMSE; deviance’ and if more than one model produced the same value for the metric, they are listed together and separated by commas.

Disease	Location	Nowcast	Short-Term	Long-Term
Campylo-bacteriosis	All countries	Alt-XGB	Alt-XGB	Alt-XGB; RF (Both); Alt-XGB
	Australia	XGB (Both)	Alt-XGB	Alt-XGB
	Israel	MLP; MLP; XGB	XGB	XGB; GLARMA; XGB
	US	Alt-XGB; GLARMA; Alt-XGB	GLARMA; GLARMA; Alt-XGB	Alt-XGB; Alt-RF; Alt-XGB
Q-Fever	All countries	RF	Alt-RF; Alt-XGB; Alt-XGB	Alt-XGB
	Australia	GLARMA	Alt-XGB	XGB
	Israel	MLP	Enc–Dec; GLARMA; Enc–Dec	Alt-XGB
	US	All Models	All Tree-based ML (Alt-XGB *)	All Tree-based ML(Alt-XGB *)
Typhoid	All countries	Enc–Dec	Alt-XGB; GLARMA; XGB (Both)	MLP
	Australia	GLARMA; XGB(Both), GLARMA; Enc–Dec	GLARMA	MLP; Alt-RF; MLP
	Israel	All Models	All Models	All Models
	US	MLP	All Tree-based ML	RF; MLP; RF

* Smallest error range. Note: “Both” refers to the Alt and Not versions of the same model.

**Table 3:** The top-performing disease models by data split. For the model, if MAE, RMSE, and deviance matched, there is one model listed; if the best model by metric did not match, the cell contains ‘MAE; RMSE; deviance’ and if greater than one model produced the same value for all the metrics, they are listed together and separated by commas.

		Number of Cases		Country Over All Months			Forecast Time Over All Locations		
Disease	Overall	High Cases	Low Cases (Zero)	Australia	Israel	US	Nowcasting	Short Term	Long Term
Campylo- bacteriosis	Alt-XGB	Alt-XGB	GLARMA	Alt-XGB	XGB (Both)	GLARMA, Alt-XGB	XGB	XGB	RF(Both); RF(Both); Alt-XGB
Q-fever	Alt-XGB	Alt-XGB	Tree-based, DL	Alt-XGB	GLARMA; Enc–Dec; Alt-XGB	All	RF	Tree-based	Alt-XGB
Typhoid	Alt-XGB	GLARMA	All	Alt-XGB; GLARMA; GLARMA	All	RF	Enc–Dec	Alt-XGB; GLARMA; XGB (Both)	MLP

Note in this table that Both refers to the Alt and Not versions of the same model.

**Table 4:** Summary of case count data used in the analysis by country and disease name for the study period of January 2009 through August 2018.

Country	Campylobacteriosis		Q-Fever		Typhoid	
	Date Range	# Regions	Date Range	# Regions	Date Range	# Regions
Australia	2009–2018	8	2009–2018	8	2009-2018	8
Finland	2009–2017	18	NA	0	NA	0
Israel	2012–2018	6	2012–2018	6	2009–2018	6
Japan	NA	0	2012–2017	47	2012–2017	47
Norway	2009–2017	18	2009–2017	18	2009–2017	18
Sweden	2009–2017	21	NA	0	2009–2017	21
United States	2015–2018	51	2009–2018	51	2009–2018	51

“NA” means no data available in EpiArchive for the specified diseases and countries. “#” means number of.

**Table 5:** Explanatory variable data types, website for public access, individual features by name, geographic location, geographic resolution, time period, and periodicity.

Data Type	Website	Individual Features	Geographic Location	Geographic Resolution	Time Period	Periodicity
Case Counts	epiarchive.bsvgateway.org accessed on 28 May 2019	Incidences of select human diseases.	Countries of interest	Region-level	2009–2018	Daily
Political Borders	gadm.org accessed on 28 May 2019	Geopolitical borders (country and within country)	Countries of interest	Region-level	2018	Single instance
Climate	disc.gsfc.nasa.gov; earthdata.nasa.gov accessed on 28 May 2019	air temperature, humidity, precipitation, soil moisture, and wind speed	Global	Gridded 0.25° × 0.25°, 1° × 1°	2012–2018	Monthly
Gross Domestic Product	www.ers.usda.gov accessed on 28 May 2019	Gross Domestic Product	Global	Country-level	Varies	Yearly
Elevation	www.diva-gis.org accessed on 28 May 2019	Digital Elevation Map	Global	43,200 × 17,200 (30 arc seconds)	NA	NA
Mortality	www.who.int accessed on 28 May 2019	Deaths by country, year, sex, age group, and cause of death.	Global	Country-level	2009–2018	Yearly
Municipal waste	stats.oecd.org accessed on 28 May 2019	Municipal waste generation and treatment	Countries of interest	Country-level	2009–2017	Yearly
Socio-political and Physical data	www.naturalearthdata.com accessed on 28 May 2019	Country and internal administrative borders; socioeconomic and political attributes	Global	Varies by country; 1: 10 m–110 m	2019	Single instance
Population	population.un.org accessed on 28 May 2019	Population by age intervals by location	Global	Country-level	2009–2015	Every 5 years
Population Density	sedac.ciesin.columbia.edu accessed on 28 May 2019	Population density	Global	30 arc-seconds	2009–2015	Every 5 years
Water Potability and Treatment	stats.oecd.org accessed on 28 May 2019	Freshwater resources, available water, wastewater treatment plant capacity, surface water	Countries of interest	Country-level	2009–2017	Yearly

“NA” means no data available.

## Data Availability

All data used in the study are publicly available with website links in the Methods section, including Table 5.

## Abbreviations

**In-Text Abbreviation**	**Description**
-Alt	a model trained without country-region as a feature
ARIMA	auto-regressive integrated moving average
ARIMAX	auto-regressive integrated moving average with exogenous variables
DE	encoder-decoder model
Deviance	Poisson deviance
DL	deep learning
DLR	dynamic linear regression
GLARMA	generalized linear autoregressive moving averages
GRU	gated recurrent unit
MAE	mean absolute error
ML	machine learning
MLP	multi-layer perceptron
RF	random forest
RMSE	root mean squared error
RNN	recurrent neural networks
SARIMA	seasonal auto-regressive integrated moving average
SIR	susceptible, infectious, and removed
SVM	support vector machines
XGB	extreme gradient boosted trees
